# Ecological Perspectives on Aging

**DOI:** 10.1111/acel.70308

**Published:** 2025-12-04

**Authors:** Alexei A. Maklakov, Monty A. Montano, Owen R. Jones, Dan H. Nussey

**Affiliations:** ^1^ School of Biological Sciences University of East Anglia Norwich UK; ^2^ Editor‐In‐Chief, Aging Cell USA; ^3^ Department of Biology University of Southern Denmark Odense Denmark; ^4^ Institute of Evolutionary Biology University of Edinburgh Edinburgh UK

## Abstract

Controlled settings may offer limited insight into the complexities of aging in natural and variable ecosystems. Artwork by Zahida Sultanova.

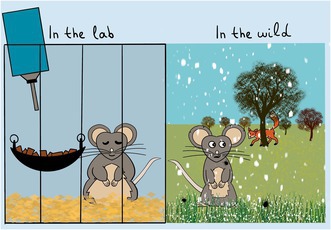

## 1 Introductory Comments

Much of our foundational knowledge in biological aging has been shaped by controlled laboratory studies, low genetic heterogeneity, and carefully constructed clinical trials. These approaches have been instrumental in uncovering key proximate cellular and molecular mechanisms that influence aging. However, their controlled settings offer limited insight into the complexities of aging in natural and variable ecosystems, and why (and how) aging patterns vary so widely across individuals, populations, and species. In contrast, a growing body of research focuses on aging in wild populations. These field‐based studies, while logistically more challenging, have a broader taxonomic range and capture the diversity of environmental conditions that organisms naturally experience. These studies therefore offer a critical context that laboratory studies alone cannot provide. Additionally, species differ markedly in many relevant characteristics, including social systems that may profoundly influence how aging evolves and is expressed.

The two research traditions of laboratory and field‐based studies have historically remained somewhat siloed (with notable exceptions), despite being grounded in a shared underlying evolutionary theory. Yet there is much they can learn from one another. Bridging this gap is essential for a more complete understanding of how aging is shaped by interactions between intrinsic mechanisms and extrinsic environmental pressures.

To bridge this gap, an alliance between *Aging Cell* and *Advanced Science* will create a special collection of both existing and invited research that incorporates ecological perspectives into aging, and the use of innovative technologies to study aging in natural populations. This initiative aims to enhance our understanding of aging trajectories in response to naturally encountered biotic and abiotic stressors over the life course. Below, we highlight thematic areas as examples underscoring the need for further research on aging in complex natural environments.

## 2 Ecology and the Evolution of Aging

Aging evolves because the force of natural selection declines with advancing age (Hamilton 1966; Charlesworth 1980; Moorad 2014). Environmental conditions shape the force of selection along the life cycle and thus shape genetic variation in aging via non‐mutually exclusive evolutionary processes (Maklakov and Chapman 2019; Lemaître et al. 2024). The weakening of selection with age can allow deleterious mutations whose fitness effects are concentrated in late life to accumulate (mutation accumulation, MA) (Medawar 1952). It can also result in selection for genes with positive effects on fitness in early life, but detrimental ones in later life (antagonistic pleiotropy, AP) (Williams 1957). At a physiological level, the disposable soma theory (DST) of aging proposes that aging evolves via trade‐offs in resource allocation between reproduction and survival (Kirkwood 1977). Here, AP genes are viewed as shaping the trade‐off between early reproduction and long‐term physiological function and weakening selection with age means that genes enhancing early reproductive performance are favored despite costs to function later in life (Lemaître et al. 2024). The emerging developmental theory of aging (DTA) proposes that aging evolves because physiological pathways (e.g., gene expression dynamics and developmental programs) optimized by selection for function in early life become poorly optimized for later life function as the force of selection wanes (Maklakov and Chapman 2019; Lemaître et al. 2024). While there is plenty of evidence to support these evolutionary ideas from laboratory model systems (Flatt and Partridge 2018), how they perform under more challenging, natural environments across species is crucial to establish their generalizability. A growing number of studies of wild birds and mammals have now used quantitative genetic approaches to provide support for some of the predictions of AP and MA in the wild (Charmantier et al. 2014; Lemaître et al. 2015). Longitudinal field studies have also identified support for early versus later life trade‐offs (e.g., between growth or early reproduction and later survival) across a range of vertebrate systems (Lemaître et al. 2015). While this is consistent with predictions of DST, there remains little evidence directly implicating resource allocation trade‐offs which is a central tenet of DST theory. Furthermore, no study has tested specific predictions of DTA in the wild to date (Lemaître et al. 2024).

Understanding aging through the lens of natural selection provides critical insights into how genetic adaptations to changing environments may influence longevity and health span. Laboratory studies often isolate genetic factors without considering the dynamic interplay between genetics and environmental factors. It is very likely that the processes discussed above, which typically implicate an important role for early life function and fitness in the evolution of aging, are highly dependent on environmental conditions. How selection declines with age and how genes shape early versus later life fitness will depend on habitat conditions, resource availability, infection and immunity, and other stressors. Laboratory studies have barely considered the role of the environment (but see Wit et al. 2013; Carlsson et al. 2021), although there is mounting evidence that the effects of genes on aging and lifespan change when the lab environment is modified (e.g., via dietary restriction) (McCracken et al. 2020; Pallares et al. 2023). Such environmental effects on age‐specific selection and gene expression are likely central to our understanding of why individuals, populations and species vary in their lifespan and aging.

Efforts to study environmental effects on aging in the wild remain in their infancy, but many long‐term field studies collect the genetic, environmental, life history, and fitness data that will enable future research (Nussey 2025). Laboratory‐based research can play an important complementary role in our understanding of the evolution of aging in natural environments. A powerful and thus far underused approach lies in combining field studies with common garden laboratory experiments to understand the interplay between ecology, evolution, and aging. For example, studies of guppies and garter snakes have demonstrated that individuals from populations that have evolved under different ecological pressures have evolved different life histories and aging patterns when compared under controlled laboratory conditions (Reznick et al. 1990; Robert and Bronikowski 2010). Further, testing the role of important genes and pathways that appear to regulate aging in model organisms under laboratory conditions under more naturalistic environments is a crucial step to establishing their generality. Recent studies have begun to do this by altering the laboratory environment of nematode worms (Carlsson et al. 2021; Sultanova et al. 2025), comparing genetic variation across wild populations of fruit flies (Schmidt et al. 2000) and investigating the performance of genetically modified mouse strains in semi‐natural enclosures (Giorgio et al. 2012). Complementing this, field studies are increasingly able to measure biomarkers of laboratory‐identified aging pathways and investigate how these are shaped by variation in the environment and how they are, in turn, shaped by natural selection in the wild (e.g., IGF‐1 levels: Lewin et al. 2017; Lodjak et al. 2018).

## 3 Aging and Stressors

Individuals vary enormously in the onset and pattern of aging they display, and the cumulative effects of past experience in terms of environmental challenges and stressors are increasingly thought to play an important role in this observed variation in late‐life health (Belsky and Baccarelli 2023). In natural environments, aging can be influenced by various concurrent ecological stressors, including temperature fluctuations, food scarcity, exposure to pathogens, and physical and/or psychosocial challenges. There is mounting evidence that early life adversity shapes lifespan and demographic aging in wild animals, although the vector of this effect is still poorly understood and may depend on the duration, severity and the type of adversity (Cooper and Kruuk 2018; Spagopoulou et al. 2020; Patterson et al. 2023). A recent study found that epigenetic aging in mice is significantly influenced by the environment, noting that mice living in more natural, outdoor enclosures exhibit different aging trajectories than those kept in controlled laboratory conditions (Zipple et al. 2025). Long‐term studies of natural populations, and especially those with comparator laboratory or highly controlled populations, offer valuable insight into how environmental experiences across the life course shape the aging process.

Two major challenges here involve identifying and measuring the relevant environmental stressors and identifying markers and assays capable of capturing variation in the aspects of biological function that matter for fitness in the wild. Recent studies have begun tackling the first challenge, testing suites of relevant environmental variables that impact natural populations and addressing the degree to which lifespan and aging are shaped by cumulative stress, early life adversity and conditions in later life (Gicquel et al. 2022; Ortiz‐Ross and Blumstein 2024; Drake et al. 2025) At the same time, many field studies are investigating how well‐established markers of biological aging developed in laboratory and human studies change with age under natural conditions, for instance using telomere length (Remot et al. 2022) methylation clocks (Le Clercq et al. 2023) and markers of immune function (Peters et al. 2019). Studies that can combine a detailed understanding of the environmental pressures facing wild animals with biomarkers of biological aging are likely to provide valuable insights into how the environment from conception onwards shapes different aspects of the aging process and contributes to variation in lifespan across the tree of life.

## 4 Multi‐Modal Data Capture

As discussed, while lab‐based research on model organisms like *C. elegans* and *D. melanogaster* has provided valuable insights into aging mechanisms, these studies often fail to capture the ecological complexity of natural environments. Existing and emerging approaches in aging research (Brenna et al. 2020), including non‐invasive biomarker sampling (e.g., from feces, hair, or saliva), remote sensing and tracking devices, and environmental genetic sampling enable researchers to monitor dynamics in natural environments. Species and organ‐adapted epigenetic clocks (Robeck et al. 2021; Sehgal et al. 2025) enable real‐time assessment of biological aging in varying environments. AI and machine learning computational methods should enable the integration of multimodal data (Callier 2022) to evaluate aging trajectories in the context of extrinsic stressors in natural environments. Wearable biosensors and activity trackers are increasingly enabling the continuous, non‐invasive monitoring of physiological and behavioral data in free‐living humans (Chen et al. 2023). By capturing high‐resolution data in situ, these technologies facilitate the study of aging trajectories under free‐living conditions, bridging the gap between controlled laboratory insights and ecologically valid human aging processes. Such approaches promise to uncover previously inaccessible interactions between environmental exposures, lifestyle factors, and biological aging pathways.

## 5 Social Behavior, Experience, and Aging

Commonly used model organisms like *D. melanogaster* and *C. elegans* show limited sociality under laboratory conditions, and even moderately social species such as mice do not exhibit the complex, sometimes highly cooperative social structures found in natural settings across the animal kingdom. Most vertebrates (and many invertebrates) show dynamic social interactions ranging from small or pair groups to complex fission‐fusion societies to cooperative group living. Sociality has emerged as an important factor in the shaping of adult health and aging trajectories. At the species level, comparative studies find that highly social species typically have longer life spans than solitary ones (Salguero‐Gómez et al. 2024). Studies across human and nonhuman mammals indicate that individuals with higher social rank and more or closer affiliative bonds with others are longer lived and display higher fitness (Snyder‐Mackler et al. 2020). Laboratory studies poorly reflect the complexity of natural social behavior, as laboratory model systems tend to show simple social systems and naturalistic behavior and available social interactions are necessarily limited by the laboratory environment and experimental constraints. Studies in the wild therefore offer a critical window onto how natural behavior interacts with genetics and the physical environment to shape variation in healthy aging.

In humans, the number of social contacts declines with age, and we observed increased selectivity and investment in interactions with a reduced social network in later life. Recent field studies have shown that similar patterns can be observed in wild primates and, strikingly, that shrinking social network size can also be observed in deer and marmots (Siracusa et al. 2022). It seems the process of ‘social senescence’ observed in humans may be a much more general biological phenomenon, although this requires further study. Whether the drivers of social decline are similar across species remains unclear, and no study to date has established a link between social senescence and reduced health or fitness in the wild. Alongside this, there is fast‐growing evidence that old individuals may play a vital role in the societies and viability of wild populations (Kopf et al. 2025). Human activity often directly or indirectly targets such highly experienced individuals disproportionately to other groups, raising calls for ‘longevity conservation’ as a management practice and further research to understand the role that older individuals play in supporting the environmental resilience of natural populations. The importance of social senescence versus experience and “wisdom” in regulating natural populations may itself be dependent on the social system of the species in question and the prevailing environmental conditions (or human stressors) the population faces. Studies in the wild can both widen our understanding of how sociality shapes aging (and vice‐versa) outside the laboratory and advance our ability to understand and manage how environmental change is likely to impact natural populations.

## Author Contributions

All authors contributed equally to this work.

## Funding

The authors have nothing to report.

## Conflicts of Interest

The authors declare no conflicts of interest.

## Data Availability

Data sharing not applicable to this article as no datasets were generated or analyzed during the current study.
